# A qualitative evaluation of the pathway for eating disorders and autism developed from clinical experience (PEACE): clinicians’ perspective

**DOI:** 10.3389/fpsyt.2024.1332441

**Published:** 2024-04-04

**Authors:** Zhuo Li, Chloe Hutchings-Hay, Sarah Byford, Kate Tchanturia

**Affiliations:** ^1^ Department of Psychological Medicine, Institute of Psychiatry, Psychology, and Neuroscience, King’s College London, London, United Kingdom; ^2^ National Eating Disorders Service, South London and Maudsley National Health Service (NHS) Foundation Trust, London, United Kingdom; ^3^ King’s Health Economics, Department of Health Service and Population Research, Institute of Psychiatry, Psychology, and Neuroscience, King’s College London, London, United Kingdom; ^4^ Tbilisi State Medical University, Psychological Set Research and Correction Center, Tbilisi, Georgia

**Keywords:** eating disorder, comorbidity, autism, adaptation, clinician interview

## Abstract

**Introduction:**

The Pathway for Eating disorders and Autism developed from Clinical Experience (PEACE pathway) is a clinical pathway of adapted treatment for individuals with eating disorders and autism in the UK. This study aims to investigate multidisciplinary clinicians’ views of the strengths and challenges of PEACE pathway adaptations, while identifying areas where further improvement is needed.

**Method:**

Semi-structured interviews were conducted with 16 clinicians who worked on the PEACE pathway. Themes relevant to the benefits, challenges and areas of improvement were identified, and a thematic map was produced.

**Results:**

PEACE Pathway brought clinical benefits such as improved understanding of patients’ perspective, improved flexibility and individualisation in clinicians’ approach, increased patient engagement, and provision of resources that are helpful to all patients with or without autism. Benefits to the service included increase in autism awareness, clinicians’ confidence, and team collaboration. Challenges were also identified, including difficulties in incorporating autism adaptations into existing treatment protocol, implementing PEACE at different levels of care, staff schedule conflicts, and increased pressure to meet patients’ needs. Overall, there is a need for systemic improvement in aftercare and community support for autism, more suitable autism screening tool, and more structured guidelines for making adaptations.

**Conclusions and implications:**

PEACE Pathway has brought clinical and service benefits, while also bringing practical challenges rooted in the difficulty in distinguishing between autism and eating disorder in comorbid population. Future areas of improvement are highlighted for PEACE resources as well as in the national support system for autistic individuals.

## 1 Introduction

In recent years, there has been a growing interest in the marked overlap between autism and eating disorders (ED) in adults (1, 2), with studies estimating the mean prevalence rate of autism in ED populations to be 23% (3, 4). It has been proposed that co-occurring autism and ED can lead to increased social impairment (5), depression and anxiety symptoms (6), longer admissions in ED treatment services (5, 7) and poorer treatment outcomes (8, 9). Therefore, there is an urgent need for ED services to effectively identify this patient group and ensure that their needs are supported. Individuals with both conditions themselves also highlight the importance of adapting ED treatment to take characteristics of autism into account (10–12).

The Pathway for Eating disorders and Autism developed from Clinical Experience (PEACE pathway) (13) is, to our knowledge, the first clinical pathway of adapted treatment for adults with ED and autism in the UK (14). The pathway was developed following needs assessment with all stakeholders including clinicians (15), carers (16), and patients themselves (10). These early studies highlighted needs for environmental adjustments, clinician education and training in autism, refeeding programme adaptations to accommodate sensory sensitivities, tools to address communication difficulties and improve patient engagement, and improved recognition and understanding of autism within ED services.

In an attempt to respond to these concerns, the South London and Maudsley (SLaM) NHS Foundation Trust National Eating Disorders Service piloted the implementation of the PEACE pathway (13), introducing adaptations such as autism screening, environmental changes including refurnishing and decoration of the service to ensure a more sensory-friendly environment, sensory tools and psychoeducation about sensory sensitivity, clinician training on autism assessment and adapting therapeutic modules and language for autistic people, development of an alternative menu, and communication support to aid communication between patients and the treatment team.

The SLaM ED service primarily serves adult women, a demographic in which autism is frequently underdiagnosed. Recent research using primary care data has indicated significant levels of underdiagnosis in adults, particularly among older age groups in the UK (17). Furthermore, previous studies have identified a gender gap in autism diagnosis, highlighting that women and girls who meet criteria for autism are at a high risk of not receiving a diagnosis (18). This disparity may be attributed to differences in behavioural characteristics compared to males (19) and a greater likelihood of camouflaging in women and girls (20). PEACE adopted a trait-focused approach in this patient group to prevent the exclusion of underdiagnosed patients whose needs would otherwise go unrecognised. This was also due to practical concerns given the long NHS waiting time for formal diagnostic assessments. As part of the PEACE Pathway, all admissions at SLaM ED service are screened using the Autism Spectrum Quotient short version (AQ-10; 21, 22) questionnaire for autistic features. In some cases where there are uncertainties about a possible autism presentation, follow up measures are used; the team would enquire more about family history of autism, developmental milestones, further observe the patient’s presentation at the service, and/or follow up with the Social Responsiveness Scale 2nd Edition (SRS-2; 23) to investigate their difficulties in more depth. Adaptations are made for those who have high autistic features, identified through screening, as well as those with confirmed past diagnoses. In line with the views and preferences of the autism community (24), identity-first language (i.e., ‘autistic person’ rather than ‘person with autism spectrum disorder’) is used in this study.

To ensure consistent implementation of the adaptations, PEACE also introduced regular ‘huddle’ meetings to facilitate communication and case discussions between the multidisciplinary health professional teams at the ED service (25). Preliminary evaluation of survey feedback has shown that 92% of trained clinicians agreed that their knowledge and skills improved and 97% agreed that the training sessions should be recommended to other ED clinicians (13). It is important that the practicalities and challenges in PEACE implementation are fully explored before trialling similar adaptation pathways at more ED services. This study, therefore, aims to investigate the feasibility of PEACE through interviewing the clinical team about their experience of implementation.

When introducing adaptations to evidence-based interventions, transparent reporting of what does or does not work is essential, to ensure that the adapted intervention is acceptable, feasible and maximises benefits for patients. Seeking feedback from clinicians is critical in this process to gauge acceptability, the degree of adaptation required and sustainability of the adaptation (26). In the development of PEACE pathway, qualitative feedback from stakeholders is regularly consulted to ensure that the adaptations made are acceptable and appropriate (10, 15, 16). However, adapting an intervention is often a dynamic process, as the context in which adaptations are made constantly changes (27). In this study, five years into the implementation of PEACE, we investigated multidisciplinary clinicians’ views of the PEACE pathway adaptations to gauge their feasibility and sustainability. Specifically, we investigated their thoughts about the following:

- Objective 1: Benefits of the PEACE pathway;
- Objective 2: Challenges in implementing the PEACE pathway;
- Objective 3: Areas where further improvement is needed.

## 2 Methods

### 2.1 Participant selection

Semi-structured interviews were conducted with multidisciplinary clinicians who worked at the SLaM adult ED service between 2017 and 2022, when the PEACE Pathway was developed and implemented at the service. A meeting was conducted first with the principal investigator of the PEACE Pathway (KT) to identify clinicians working at the service during this time period with good knowledge and involvement with the PEACE Pathway (i.e., participated in PEACE Pathway training and regular meetings). A list of potential interviewees with varied roles representative of the multidisciplinary team was identified (for example, counselling psychologists, consultant psychiatrists, psychology assistants, dietitians, family therapists, and occupational therapists). All potential interviewees identified were invited by the lead author (ZL) by email to participate in the study. The invitation email explained the purpose of the study and that clinicians were invited based on their involvement with the PEACE Pathway. Clinicians who expressed interest then received an information sheet and a consent form to be signed if they agreed to be interviewed. Written consent was acquired prior to interviews, including consent for the interview to be recorded. Ethical clearance for this project was granted by King’s College Research Ethics Committee (MRSP-21/22-28800).

### 2.2 Interview

Participants were interviewed by ZL face to face or online, depending on clinicians’ preference. During the interview, a topic guide was used to ask participants the following questions:

1. Could you tell me about your involvement with the PEACE Pathway? (Gatekeeping question to gauge participant’s involvement and identify focus points for follow-up questions)
  - Follow up: did you find the [PEACE component that the participant mentioned in their reply to Question 1] helpful or unhelpful? How?
2. Was there anything from the training that really stuck with you? (To gauge participant’s exposure to PEACE Pathway training)
3. Have you used any other PEACE resources during clinical practice? How helpful or unhelpful did you find them?
4. Do you have any suggestions for how the PEACE Pathway can be improved?

The interviewer also used follow-up questions asking for further details and examples after asking the main interview questions. All interviews lasted between 30 minutes and one hour. Recordings of the interviews were then transcribed verbatim by ZL with all identifying information removed at the point of transcription. The interviews continued until no new information emerged, indicating data saturation.

### 2.3 Analysis

Interview data were analysed in NVivo 12 using thematic analysis (28, 29). Firstly, transcripts were read and reread by ZL and CHH for content familiarisation. A coding framework was developed deductively based on the research objectives and topic guide questions. Transcripts were read and reread by ZL and CHH, and the coding framework was further refined based on data content, agreed by all authors, and applied to the data by ZL. Preliminary themes emerged from analysis of the coded data, which were then reviewed and modified by scrutinising the data associated with each theme in the context of the entire data set. Finalised themes were re-worded for clarification where appropriate. The relationships between themes and subthemes were checked for overlap. All results are reported according to the Consolidated Criteria for REporting Qualitative studies (COREQ) checklist (30).

## 3 Results

In total, 16 clinicians were approached, consented and interviewed before data reached saturation. All clinicians worked at the SLaM ED service where PEACE Pathway was implemented. The sample represented the multidisciplinary team structure including a range of professions: assistant psychologists, clinical psychologists, consultant psychiatrists, dietitians, family therapists, and occupational therapists. Clinicians all had experience treating patients with co-occurring ED and autism. Among the participants, 12 (75%) were female and 4 (25%) were male.

**Figure 1:**
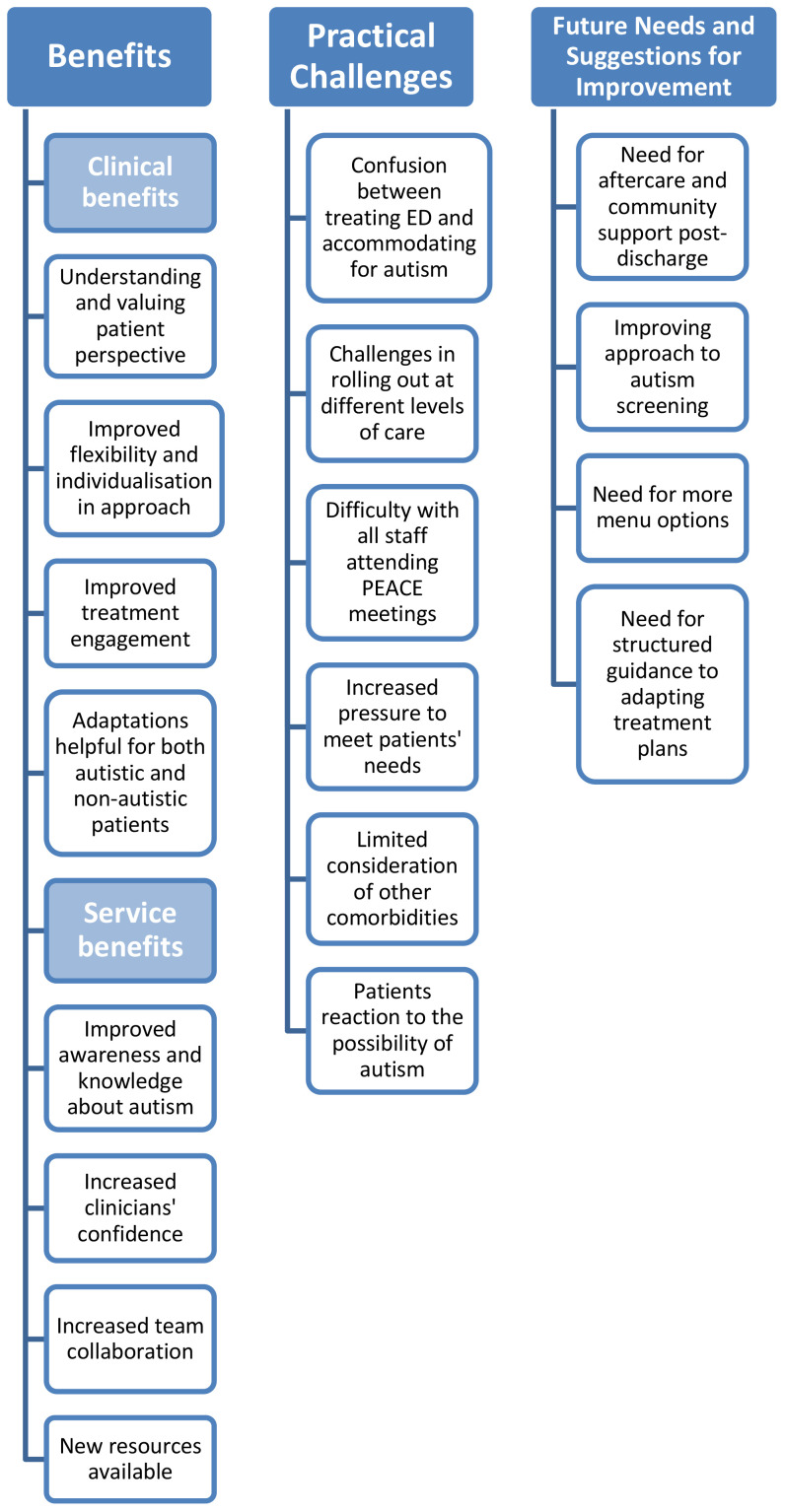
Thematic map of clinicians’ views.

Three main themes emerged from the analysis: benefits of the PEACE Pathway (including clinical benefits and benefits to the service), practical challenges, and future needs and suggestions for improvement. All themes and sub-themes are presented in a thematic map (**Figure 1**). Key findings are reported below, supported by participant quotes. All quotes are anonymised with participant numbers.

### 3.1 Clinical benefits

Clinicians described several clinical benefits of the PEACE Pathway on their treatment approaches. Firstly, they highlighted the collaborative nature of the pathway development and how this helped them understand and value patients’ perspectives. Patient involvement in the decision-making process was highlighted as particularly beneficial in the earlier stages of pathway development. Clinicians described seeking patient feedback on their preferred sensory adaptations (e.g., introducing silent key caps to reduce the sound of the ward keys and decluttering ward environment), communication needs (e.g., designing and giving out the communication passport for patients to fill in and having conversation cards available in the public area on the ward), and preferred colour scheme for environmental adaptations.

“This wasn’t, sort of, ‘okay, this is a clinician project’, or ‘this is a carers’ [project]’, this was our project in terms of the patients, the carers and the clinicians all working together in deciding and making sure they had a voice about how would they want the sort of dining room, or the sort of corridors to kind of be. I remember even doing the psychology board with them, and we’ve kind of done that all together, as well, sort of during Christmas break that time, designing it together. So, making sure that they were very involved.” – Participant 3

“We talked to them about kind of their communication style, what kind of-, how do they seek support, what they find helpful, not helpful and tried to adapt it in such way.” – Participant 4

Participants also described improved flexibility and individualisation in their approach to meeting unmet needs of patients. Firstly, this was reflected in clinicians creating autism-friendly version of documents that were given out, such as colour-coding menus or adding bullet points and using concise language on information pamphlets and post-session summaries. Secondly, adaptations were made on an individual basis during sessions, such as adjusting environmental settings (e.g., lighting or seating positions), using clearer language and reducing metaphors, giving advanced notice about changes taking place, covering fewer topics in one session, as well as adjusting the structure and pace of the sessions (e.g., having shorter sessions that are more frequent). Participants also highlighted the importance of clarifying patients’ sensory needs and communication needs at the start of treatment using PEACE resources such as the communication passport (31) to ensure that appropriate adaptations are made. Thirdly, participants described being able to tailor treatment goals and make informed clinical decisions based on patients’ autistic features. This involved establishing realistic expectations of what patients can achieve in treatment, and how the care plan can be adjusted to accommodate patients’ needs while ensuring safe recovery from ED. For patients whose autistic needs posed challenges to treatment, some clinicians would share information from the PEACE book and work collaboratively with patients to establish treatment priorities, such as asking questions like “What is your priority for change?” and “What do you actually want to work on?” while also considering what is realistic for the individual.

“And it’s so easy to do, it takes like, three, four minutes just to, you know, turn the lights off, open the blinds, or close the blinds, or windows, you know, either sit side by side, lots of people like that, rather than like giving all that eye contact. And yeah, just like the length of the session, you know, some people wanted shorter sessions, some people wanted more regular sessions, some people wanted less regular [sessions]. So, you could be a little bit more bespoke.” – Participant 10

“For example, in one case, we talked about, you know, sensory and taste difficulties and also other hypersensitivities the patient had, for example with the sunlight, and that we made some adaptations like closing the curtains. We also discussed whether she should be allowed to go on walks in the evening when it was already quite dark. Yeah, so that was balancing the autistic needs and the safety of the patients and also health aspects as this patient needed vitamin D supplementation because she didn’t have enough sunlight.” – Participant 5

Improvement in treatment engagement was also noted, with some participants suggesting that this occurred after patients’ needs were met. Clinicians gave examples of patients who were disengaged in the past but came into treatment more once their needs were met by individualised support, and of patients who were able to go on to more complex psychological work with appropriately adapted communication methods. Novel adaptations that led to better engagement were often shared in case discussions at PEACE meetings to ensure other staff were also aware of the approach, and could use it in their practice.

“[The patient] never engaged in psychological therapy, [ … ] because: one, her, she said her needs weren’t being met; and also it was rather, sort of sensory sensitivities, she was experiencing. [ … ] So we were making sure that we were thinking about things much more holistically. [ … ] It was a lot of adaptations, she engaged in all our sessions together. And even after that, we were able to kind of go on to more complex psychological work with her such as, like, cognitive behavioural therapy, because she felt like we were listening and her needs were being met.” – Participant 3

“[The patient] hated being in the room and found it really daunting. So I sort of tried to think outside the box and I said, let’s do an experiment. And we’ll do like a telephone conference. So we can all dial in. And then that will take away some of like the social, overwhelming social nature of it. I mean, it was very interesting because he loved it. And he really got a lot out of it and kept coming back as he disengaged in the past.” – Participant 11

Clinical benefits of PEACE Pathway adaptations were not limited to autistic patients only. Some of the resources such as the communication passport and sensory tools are available to all patients admitted to the service, and this was helpful as even patients without autistic features can have communication preferences and/or sensory needs. It was also highlighted that sensory adaptations were particularly useful for trauma informed approaches, as sensory sensitivity is commonly seen in patients who had experienced trauma.

“Everyone is different and everyone has different sensory needs, whether you’re on the autistic spectrum or not.” – Participant 4

“We wanted it to be a peaceful calming environment for, for everybody. And I think that’s the important thing – it is for everybody because we know that … would suit an awful lot for the folks we work with eating disorders, not just those with a diagnosis of ASD.” – Participant 7

### 3.2 Benefits to the service

Participants also reflected on PEACE Pathway’s impact on the service overall, and highlighted improvement in the clinical team’s knowledge, skill, and awareness of autism in their daily practice. Many participants mentioned the benefits of attending PEACE training sessions, which covered a wide range of topics, including autism assessment and formulation, which helped clinicians identify autistic features such as camouflaging that could interfere with treatment, and therapeutic and environmental adaptations for autism, which supported the development of strategies for individualising treatment approaches. Moreover, participants mentioned that PEACE raised the level of autism awareness across the team, and it was easier to have autism-related discussions when the team overall became better informed about autism.

“We would have a lot of experts in the field, come in and teach us and train us, whether that was to do with formulations, adapting CBT, it was all so helpful.” – Participant 3

“The fact that the team as a whole was becoming better informed about autism, it was really helpful because then there were various sort of places that you could go, people that you could discuss things with.” – Participant 6

With enhanced knowledge and skills, clinicians felt a greater sense of confidence and credibility when working with autistic individuals. Consequently, they felt more at ease engaging in open, transparent discussions about autism when necessary. This increased willingness to address autism-related topics contributed to the improvement of therapeutic relationships and fostered greater trust.

“I feel more confident working with people with autism because I have that background. And I also think I’m more credible to working with them.” – Participant 7

Participants highlighted PEACE Pathway’s positive impact on team collaboration. This was mainly achieved through PEACE huddles, which are brief, regular meetings for case discussions and updates joined by clinical teams across the ED service. It was described as a valuable forum for hearing different perspectives from multidisciplinary team members, collective problem solving, and sharing formulations and dilemmas about patient cases.

“It really brought the team together across the services. You’ve got people from day care, from outpatient, inpatient, step up.” – Participant 11

“It was also a good space to know about what was happening where, because like I said, I’m a little bit out of touch with what’s happening on the ward. But that was a place where I could find out what they were doing, and vice versa.” – Participant 13

Lastly, many participants highlighted that the PEACE Pathway has made available a wide variety of resources to use flexibly in practice. Participants appreciated the low stimulus room, the autism-friendly introduction packages for patients, the sensory psychoeducation materials, and the communication passport. The PEACE website (peacepathway.org) and PEACE book (32) were also highlighted by most participants as good sources of information for themselves as well as for signposting patients and families to.

“I think there’s some useful information and signposting on there. And I know [ … ] from having discussed with patients that they found it helpful. They’ve used it and have spoken about it in therapy. One patient in particular said that they wish this had been, this had been known sooner.” – Participant 10

### 3.3 Practical challenges

Many participants described their dilemma between treating ED and accommodating for autism. For example, it can be difficult to disentangle which symptoms arise from the core symptoms maintaining ED or difficulties associated with autism. This complexity is particularly evident when patients’ restrictive behaviours are influenced by factors from both conditions, such as body image concerns coupled with a preference for routine and predictability. Clinicians often worried that making accommodations for autism would inadvertently exacerbate the ED. This issue was highlighted particularly with the use of the PEACE menu, which is an alternative autism-friendly menu mainly consisting of bland tasting food with homogeneous texture that is calorie-matched with the regular menu used at the service. Food items on the PEACE menu are also photographed and pre-packaged where possible to maximise predictability and reduce patient anxiety. However, clinicians expressed concerns that some patients would choose the alternative menu not necessarily because of autism, but out of the desire to restrict because although the alternative menu is calorie-matched, some of the items may be perceived as lower calorie due to their blandness. There were also concerns that patients who chose the alternative menu all the time in order to avoid the standard menu would risk being on a restrictive and rigid diet with limited variety. Some clinicians noted that reasonable adaptations should be made in the beginning of treatment, but suggested that therapeutic challenges need to be introduced in the process to encourage improvement, for example to increase variety in food intake or to challenge rigid behaviours. Such challenges can be crucial to ensuring good transitioning into everyday life, but it is difficult to conclude from PEACE Pathway guidance when to reduce adaptations and introduce these challenges.

“In the beginning, we make a lot of adaptations to not challenge [the patients] too much with the texture of food, with the sunlight or with a, with the noise, etc. But there also needs to be some improvement and some therapeutic challenges. And it’s not always clear for me from the information I get from the PEACE huddles etc. how quick we should make improvements and challenge these things.” - Participant 5

“The [PEACE] menu doesn’t represent what’s available in cafes, and [supermarkets] and stuff like this. And I think it is difficult with the eating disorder patients who tried to elicit … using all of the adaptations, because then effectively, they’re just facilitating a very restricted diet.” - Participant 12

Clinicians’ uncertainty between ED treatment and autism adaptations was intensified when PEACE was rolled out to different levels of care, where treatment goals and service structures can vary. For example, participants noted that implementation of the PEACE Pathway was stronger on the inpatient unit than it is in the outpatient unit. Differences in treatment goals were also highlighted by participants, with inpatient services noted as prioritising weight restoration, whereas day service and outpatient teams aim to support weight stabilisation and transition into the community. As a result, clinicians in outpatient and day services tend to prioritise therapeutic challenges and may be more focused on manualised sessions rather than making adaptations. Lastly, participants pointed out differences in treatment format, where day services are essentially a group programme, which presents greater difficulties for clinical staff to support patients individually. Clinicians described that as much as they would like to tailor to individual needs, this also has an impact on the group elements of the treatment. For example, a clinician described that when one group member gets adapted treatment and is allowed to wear headphones during mealtimes, other members would be aware and would question the fairness of this. The impact of adaptations on patient dynamics in a group can be particularly challenging.

“I do think it’s such an important thing to hold in mind the comorbidity and the crossover of traits, but I guess there is also sometimes the adjustment of what can be adapted on the inpatient ward to then when they come to day services, or outpatients, [where] we are not able to meet that level of adaptation. Or we are a bit more hesitant to.” – Participant 12

“In day service, the nature of the intervention is that you are going to be raising people’s anxiety. They are confronting anxiety provoking situations around food and around emotions. And that’s the nature of the intervention. And I suppose sometimes people might say in their [communication] passport, you know, don’t, don’t say this or that to me. But that might be something that we need to speak to them about, in order to sort of challenge the eating disorder, or challenge unhelpful ways of communicating.” – Participant 13

Participants also discussed difficulties with not all staff members being able to attend PEACE huddle meetings. Despite the importance of multidisciplinary team cohesion for disseminating clinical innovations to team members, participants highlighted the struggle for all disciplines to attend the meetings regularly given their already demanding workload.

“It can be hard in terms of sort of staff time. It can be a struggle for, say, for instance, some disciplines to find the time to attend these, due to various other demands on the ward as well.” – Participant 3

In addition, clinicians mentioned that some PEACE adaptations have increased the pressure they are under to meet patients’ needs, which sometimes can be unrealistic and unhelpful in terms of recovery:

“I think, another thing about the communication passport, I would say that it needs to be used with caution, and also to think together with the patients. I don’t think it’s humanly possible for us to remember, in such fine details, some communication passports are so detailed in terms of how they like and not like to be spoken to, and so on. And to hold all these patients in mind is going to be very difficult. So I think it’s helpful to have an idea, but we also need to invite the patients to think about how can we be more flexible with it? And some patients will say in the communication passport, I don’t, I don’t ask for support. And we can’t just accept that, because that’s not going to be helpful for them.” – Participant 4

Some participants raised concerns that there can be a lot of crossover between potential diagnoses in complex cases, for example personality disorder and autism, or complex post-traumatic stress disorder and autism. Focusing prematurely on autism can lead to overshadowing of other possible diagnoses, stopping patients from getting appropriate treatments. This is exacerbated by the fact that waiting lists for a formal autism assessment in the UK are long, often exceeding two years, thus there is a danger of premature suggestions of autism.

“The PEACE Pathway – although I think it’s really good, and it has lots of resource and it has helped loads of people – I think runs the risk of having an autism bias to the extent where it gets over thought about at the cost of being able to distinguish other stuff.” – Participant 12

In addition, suggestions of possible autism are sometimes met with negative reactions from patients and families. Some may reject the idea, and some may not be prepared to receive a suggestion of autism from an ED service. Disclosing this possibility to patients and families may therefore be a stressful experience for clinicians.

“But her parents, well, her mum is very rejecting of the diagnosis and is embarrassed about it. So I think you might have, yeah, just be prepared that some people might not be as understanding. … You know, it’s been really upsetting for her. Whilst it’s like an epiphany, it’s also really upsetting because she’s like, ah, so like, I’ve got this thing, and it’s about me, and it’s with me.” – Participant 11

### 3.4 Suggestions for improvement

In the context of the challenges of and barriers to PEACE implementation, participants also reflected on potential areas of improvement in the future. The need for aftercare and community support was highlighted by many, not only as a suggestion for PEACE but more as a national urgent need for support for autistic individuals. Clinicians mentioned that the discontinuation of adaptations once patients leave the ED service is worrying. The PEACE Pathway therefore needs to be developed further, taking into account aftercare needs.

“So I think management of the aftercare needs to be improved, because you create a lot of expectations in the patients if you offer ASD and anorexia nervosa service on the ward, and the patients think this really continues afterwards. And it doesn’t. Where the patient then has identified all the problems with the therapists, but will become a bit hopeless, if those identified needs are not met, after the treatment on the ward.” – Participant 5

Participants also pointed out that the autism screening approach needs to be improved. PEACE uses screening However, participants raised several concerns about this process. Firstly, the participants cautioned against exploring autism with patients immediately after they score over the threshold on the AQ-10 without seeking further evidence. Participants pointed out that there is need for structured guidance and clear decision points for the follow up procedure after the initial AQ-10 screening, for example what additional features to look for in patients’ presentation, and when to mention the possibility of autism with patients. Secondly, participants were concerned that the AQ-10 is not accurate enough with possible overlap with other diagnoses and starvation effect. The result therefore requires careful interpretation.

“I think with women as well, [AQ-10] is not quite, I think, if you look at its psychometric properties, it’s never, the internal consistency is never that high.” – Participant 11

“So [score on the AQ-10] was just flagged up as being positive. And then it’s not as clear what do we then do with it? Often, we then decide to do something with it if we’re really struggling with management, and we thought, actually, we need to think more about autism, then it becomes kind of more, more kind of on the forefront of our mind.” – Participant 4

Clinicians also mentioned that the PEACE menu could be improved to include more options, for example a version with more vegan options. It was also noted that a version for people with sensory hyposensitivity could be developed, in addition to the current version which is for people with hypersensitivity and dislike strong smells or tastes. The hyposensitivity version, for example, could include food items that have stronger smells and tastes and would satisfy sensory seeking needs.

“I think quite a lot of that group are vegan. So if they were to be another vegan option on the PEACE menu that might actually support them having more than just one food every single day, maybe there’d be two.” – Participant 9

Some participants preferred more structured, step-by-step treatment plans from PEACE, instead of general guidance on how to adapt sessions. Two benefits were proposed for this structured approach: easier and more specific treatment planning for clinicians, and clearer structure for patients with high rigidity.

“For example, in CBT for depression, you have these manuals where you will see in, in week one, you make this formulation. Week two, you talk about positive activities. Week three you implement the first change, etc.[ … ] And we don’t have such a clear plan developed for people with ASD and anorexia. [ … ] We have identified the challenge, we have been given some guidance, but let’s say for someone who starts on the ward, very specific guidance would be good.[ … ] So, coming from general guidance to a more specific treatment plan - I think that would be helpful.” – Participant 5

## 4 Discussion

### 4.1 Overview

The findings of this qualitative evaluation highlighted a broad range of benefits of and challenges in the PEACE Pathway from the perspective of multi-disciplinary clinicians working in the SLaM ED service where the PEACE Pathway had been implemented. Before discussing benefits, challenges and areas of improvement subsequent to the implementation of the PEACE Pathway, it is useful to reflect on the areas identified in previous needs assessments conducted with stakeholders prior to and during PEACE Pathway development, which are summarised in **Table 1**. In 2017, Kinnaird and colleagues interviewed clinicians in the same SLaM ED service about their views on working with patients with ED and autism, which highlighted a lack of clinician confidence, a lack of clear pathways for autism assessment referrals, problems with patient/therapist communication, difficulties identifying and articulating emotions, and lack of systematic guidelines and staff training on adapting treatment (15). A subsequent study involved interviews with patients and found that people with co-existing eating disorders and autism struggled with the short time frames for treatment and could not engage well in refeeding due to sensory difficulties (10). The same study also highlighted the importance of involving patients in deciding how to adapt services to support patients with autism. A third study with carers raised issues such as difficulty getting an autism assessment, sensory difficulties, a need for a tailored approach to treatment and difficulty getting services to adapt treatment (16).

**Table 1:** Stakeholders’ needs based on earlier studies and themes arising from the interviews reflecting areas addressed by PEACE.

Stakeholder	Needs based on earlier studies	Areas addressed by PEACE
Clinician	Need to improve confidence supporting the co-morbidity (15)	Increased clinician confidence (also reported in: 25)
Clinician	Need to improve expertise and experience, and sharing of expertise (15)	Increased team collaboration through huddle meetings and case studies (also reported in: 25)
Clinician	Need to improve understanding of autism and willingness to adapt (15 & 10, 16)	Improved overall awareness, skill and knowledge about autism
Clinician	Need to address communication challenges (15)	Flexible and collaborative approach leading to improved treatment engagement; new resources developed (communication passport, conversation cards)
Patient	Need to develop better relationships with clinicians and to feel better understood (10)	Flexible and collaborative approach leading to improved treatment engagement
Patient	Need to feel listened to and able to influence adaptation of their treatment (10)	Collaborative approach focused on understanding and valuing the patient perspective
Patient	Need for a flexible, tailored and individualised approach (10, 16)	Improved individualisation and flexibility in treatment format, structure, tools and goals
Patient	Need to improve response to sensory and communication difficulties (10, 16)	Environmental and sensory adaptations (also see 33) and new resources available (e.g., communication passports and alternative menus)
Carer	Need for improved access to autism assessment (16)	Autism screening tools introduced as standard screening procedure for all admissions; clinicians received training in assessing and recognising autism
Carer	Need to improve support for sensory difficulties (16)	Environmental and sensory adaptations and new resources available (e.g., service environment re-designed, sensory workshop introduced and sensory toys available, also see 34
Carer	Need for a tailored approach to treatment and for service-wide adaptations to treatment (16)	Improved individualisation and flexibility in treatment format, structure, tools and goals; improved team collaboration and awareness

**Table 1** summarises stakeholders’ needs assessed by previous studies in parallel with relevant themes arising from the current evaluation. Some of the themes are corroborated with evidence from other studies (for example, 25). Themes from the interviews suggest that many of the previously identified challenges and needs were addressed by PEACE: clinicians reported an increase in their confidence; new resources, such as communication passports, alternative menus and sensory aids, have been developed and disseminated to help with communication and sensory difficulties; patients are now receiving a more tailored and individualised approach to treatment, with adjustable time frames and pace for treatment that better suits their needs; and clinician skills and knowledge about autism improved as a result of training. The PEACE Pathway also incorporated autism screening in order to meet the need for clear guidance on autism assessment; this was however both appreciated by clinicians and also highlighted as an area where improvement is still needed, discussed in more detail in section 4.3. Furthermore, some of the benefits highlighted in the current evaluation exceeded previously identified needs, for example PEACE Pathway adaptations and resources were beneficial not only to autistic patients, but to all patients with communication or sensory needs.

### 4.2 Benefits and challenges

Clinicians reported many benefits of the PEACE Pathway, such as improved understanding of patients’ perspectives, improved flexibility and individualisation in approach, increased treatment engagement, and provision of resources that are helpful to all patients, with or without autism. PEACE also brought benefits to the clinical service overall, increasing general awareness and knowledge about autism, boosting clinicians’ confidence in treating the comorbidity, providing platforms for team-wide collaboration, and making the treatment programme overall more resourceful. These findings may be linked to autistic patients’ reduced use of intensive treatment after PEACE implementation in the preliminary cost-savings analysis of PEACE (7), suggesting that PEACE clinicians are making appropriate changes to meet the needs of autistic people. Indeed, PEACE adaptations align with the recommended adaptations for working with autistic people by the NICE guidelines (35), including having breaks in therapy, increased use of written and visual information, involving carers, and avoiding metaphoric language when needed. In addition, PEACE also introduced aspects that are similar to CBT adaptations that have been tested by other studies to be clinically effective for common mental health problems in autistic people, for example adjusting the structure and pace of therapy, and including materials and skills training to enhance patients’ understanding of emotions (36, 37). A systematic review by Walters, Loades and Russell (38) found that interventions that were effective for autistic young people tended to use more modifications than those recommended by NICE. It was also found that such interventions tended to use more disorder-specific modifications i.e., tailoring to the specific psychological disorder being treated. Overall, the benefits and strengths of PEACE highlighted by clinicians in this study are encouraging.

Clinicians also identified challenges in the process of implementation, due to the difficulty in incorporating autism adaptations into existing ED treatment protocol and goals. For example, accommodating for patient’s sensory needs by providing noise-blocking earbuds during mealtimes conflicted with social eating which is encouraged by clinical teams in inpatient and day patient treatment settings. Similarly, supporting patients with nutritional rehabilitation using their preferred option of an alternative bland menu conflicted with the typical goal of increasing the variety of food choices in ED treatment. At the core of these individual-level challenges lies the difficulty of distinguishing between ED and autism, and clinicians’ hesitance to deviate from standard treatment protocols. Indeed, disentangling ED and autism can be complicated in a clinical setting, especially when certain presentations in people with the comorbidity can be influenced by factors from both conditions. For example, restrictive eating could be due to body image concerns (rooted in ED) combined with an autistic need for routine and predictability. In this case, it is difficult for clinicians to decide whether they should make adaptations. It is therefore important that adaptations are constantly discussed and formulated in supervision and clinical meetings on a case-by-case basis to ensure peer support for clinicians during decision making. Recent studies have also started to shed some light on distinguishing between common features in anorexia nervosa and autism (1, 2). Brede and colleagues (11) proposed a model of autism-related mechanism underlying restrictive eating behaviour, including how autistic traits could lead to restriction directly due to sensory sensitivities and/or autistic cognitive profile, or indirectly through increasing negative emotions which leads to restricted eating as a coping mechanism. This model is yet to be empirically tested, but it is a helpful theory for clinicians to consider. Meanwhile, a framework was recently proposed to outline clinical features associated with both autism and anorexia nervosa, also highlighting the potential differences in presentation, which can provide useful guidance in clinical practice (2). However, more detailed guidelines are needed to distinguish between autism and other types of EDs such as bulimia nervosa or binge-eating disorder, and to clarify priorities for treatment in different clinical scenarios where treatment protocols may be in conflict.

Further dilemmas were highlighted in relation to the rolling out of adaptations in day service or outpatient setting, where team structures and approaches differ from those in inpatient settings, with greater emphasis placed on patients’ flexibility and independence in preparation for full recovery. Indeed, it was highlighted that implementation of the PEACE Pathway was stronger and happened faster in inpatient settings compared to day service and outpatient settings. This may be due to the size of the team (the inpatient team is smaller than the day/outpatient teams and therefore barriers to communication may be lower), the nature of the psychological intervention (where the inpatient service provide more one-to-one interventions compared with the group based programme in the day service, which presents more difficulties for clinical team to adapt care for individual patients), limits on the number and time of outpatient sessions provided to each patient (and so it may become difficult to meet the needs of an autistic person in a fixed number of sessions), and frequency of team communications (team meetings are run almost daily in the inpatient service and weekly in day/outpatient services). As a result, adaptations that worked in an inpatient setting may be less meaningful in other levels of care.

One potential way to address this problem is to refine the implementation of PEACE in outpatient and day services to better align with treatment goals and structure. For example, given the emphasis on Cognitive Behavioural Therapy (CBT) in outpatient clinics, it may be beneficial to focus on adapting the structure of CBT sessions and language to ensure clarity and consistency (36). In the day service, which typically involve more group-based activities, it could be helpful to prioritise support aimed at making communication in groups more comfortable for autistic individuals, who may find group work difficult (14). When rolling out adaptations at different levels of care, constant tailoring, reviewing and supervision is required to align with core goals and the structure of alternative treatment settings. Appropriate evaluation is highlighted by previous research as a crucial step in this process (39), and future evaluation studies are warranted to gauge the impact of refining implementation of the PEACE Pathway in day or outpatient settings.

The development of the PEACE Pathway adopted an iterative Plan, Do, Study, Act (PDSA) methodology (13) to ensure best practice for service improvement (40), and involved collaboration with patients, clinicians and carers to ensure that all stakeholder values and needs were considered. This approach closely reflects the recommended models for making adaptations to evidence-based practice in implementation science (39, 41). Nevertheless, clinicians in the ED service are supporting patients rather than testing theoretical models, and individual patient differences create a variety of dilemmas which are unlikely to be fully resolved through research. Instead, it is likely that sustained use, testing, modification, and evaluation of the PEACE Pathway is essential to support clinicians navigating these dilemmas. This highlights the importance of providing continuous support for clinicians. More resources should therefore be allocated but not limited to: regular clinician training to improve their confidence and skills, lowering barriers to multidisciplinary team decision-making regarding how to manage the dilemmas, and establishing emphasis on value in addition to effects across clinical services.

### 4.3 Future directions

Clinicians in this study also expressed a need for more structured, preferably manualised guidance. At the ED service, clinicians are trained to use a range of structured, evidence-based ED treatment protocols in their daily practice (42–44). For treatment of coexisting disorders like ED in autistic people, however, current guidelines recommend offering interventions for the specific disorder, not for autism (35), while only listing possible adaptations for autism without specifying the order of priority or structure in which the adaptations should be made. Whilst a PEACE Pathway guide to adapting treatment for autistic people with ED has been published, which includes practical examples and guidance written in multidisciplinary perspectives (32) and was highlighted as helpful by many participants, some called for more structured, step-by-step guidance. However, it is not currently clear whether a more structured guideline would be feasible to manualise the PEACE approach, as a result of the complex interactions between the co-morbid conditions. Evident in this study was participants’ perception of contradictory benefits and difficulties. For example, the PEACE menu may reduce anxiety in patients and therefore be welcomed by clinicians providing mealtime support, but this may contradict dietitian guidance to increase food variety. Similarly, noise-reducing tools such as earbuds that aim to ease a patient’s sensory sensitivity might also create barriers for ‘social eating’, which is one of the goals of ED treatment. One strategy suggested by previous work for challenges like this is to develop complex interventions that are flexible and allow for variations (45). The structure of PEACE resembles such an intervention: it includes a wide range of resources and adaptation guidelines that can be flexibly used to tailor to the individual cases. However, this also creates barriers for clinicians who prefer structured guidance. Sustained use and testing of the PEACE Pathway, alongside development of more structured guidance, is warranted.

Clinicians in this study also expressed uncertainties about the screening procedure for autism. Prior to PEACE implementation, interviews with clinicians at the same service suggested that there was no clear pathway for ED clinicians to refer their patients for an autism assessment (15). The PEACE Pathway therefore introduced autism screening into standard practice using the AQ-10, as it is the only measure recommended by NICE for initial assessment of autism in adults (35). However, previous work has shown that the AQ-10 is not a good predictor of diagnosis in clinical samples (46). The screening tool’s poor specificity was highlighted, reflecting high rates of false positives. In addition, screening for autism in people with ED is particularly difficult, due to overlapping features between the two conditions such as cognitive rigidity and social difficulties (2). These overlaps make it more difficult for autism screening tools to distinguish between autistic features and ED symptoms. Therefore, a more suitable autism screening tool for an ED service is needed. Previous work has suggested adding subscales on auditory sensitivity, social compensation and externally orientated thinking to the AQ-10 to improve its ability to distinguish between ED and autism (47). However, this model is yet to be tested further. Another challenging aspect of the screening process is deciding how to proceed with a positive result on the AQ-10. Currently, this decision relies on the clinical expertise and experience of senior clinicians, who factor in follow-up assessments and clinical observations, including evidence of sensory sensitivities, management of social interactions, body language and eye contact, special interests, and other aspects. However, this process is not yet fully operationalised. A structured guide for decision making when a person scores on initial screening could be developed to aid this process, although it should leave enough room to consider individual differences between patient cases.

A need for improvement in aftercare for autistic people was also highlighted. This is highly relevant but exceeds PEACE Pathway’s span of influence, and rather reflects a national need for destigmatising autism and improving diagnostic pathways and community support. Over the past 20 years, there has been a 7-8 times increase in recorded incidence of autism diagnoses with the greatest rises among adults (48), yet current service provision for autistic adults is in its infancy compared to health and education services for autistic children (49, 50). The COVID-19 outbreak further increased NHS backlog of autism assessment referrals by 169% from pre-pandemic level (51, 52). This systemic gap in support for autistic people affects clinicians’ decision making in a range of areas. Some clinicians hesitate to discuss the possibility of autism with patients, knowing that resources and support become very limited once patients are discharged, and patients could spend years on the waitlist for a formal assessment of autism. Some are faced with negative reactions and denials from patients and families due to stigma on autism. Some worry that the adapted environment at the ED service inadvertently creates a gap with the ‘real world’, and once a patient is discharged, the discontinuance in autism-friendly adaptations could lead to deterioration. This affects both discharge planning and clinicians’ readiness to make adaptations. Therefore, improvement in the implementation of PEACE Pathway requires a system integrated with efficient autism diagnostic and aftercare services. Research on strategies to improve adult autism services in the UK, including assessment and diagnostic services and support networks, is currently underway (53–55).

### 4.4 Limitations

Nursing staff were not interviewed in this study due to their lower level of engagement with the development and/or implementation of PEACE Pathway. However, their feedback could be invaluable as they have direct daily contact with patients, and should be investigated by future studies focusing on gaps and areas of improvement in implementation. The heterogeneity of this study sample, however, should strengthen the credibility of the study, as participating clinicians varied in gender, age, seniority, and discipline. Another limitation is the lack of information regarding clinicians’ years of experience in treating patients with ED and those with comorbid autism and ED. Future studies should explore whether clinicians’ experience influences the acceptability of the PEACE pathway.

## 5 Conclusion

The PEACE Pathway has potential benefits for clinicians’ approach with patients and service-wide knowledge and awareness of autism, while also bringing practical challenges. Future areas of improvement are highlighted for PEACE resources as well as in the national support system for autistic individuals. This study provides initial evidence for the feasibility and acceptability of the PEACE Pathway, and warrants future studies to investigate patient experience on the pathway.

## Data availability statement

Anonymised interview data can be provided upon request to the corresponding author.

## Ethics statement

The studies involving humans were approved by King’s College Research Ethics Committee. The studies were conducted in accordance with the local legislation and institutional requirements. The participants provided their written informed consent to participate in this study.

## Author contributions

ZL: Data curation, Formal Analysis, Investigation, Writing – original draft, Writing – review & editing. CH-H: Formal Analysis, Writing – review & editing. SB: Supervision, Writing – review & editing. KT: Supervision, Writing – review & editing.

## References

[1] Westwood H Tchanturia K. Autism spectrum disorder in anorexia nervosa: an updated literature review. Curr Psychiatry Rep. (2017) 19:41. doi: 10.1007/s11920-017-0791-9 28540593 PMC5443871

[2] Kinnaird E Tchanturia K. Looking beneath the surface: Distinguishing between common features in autism and anorexia nervosa. J Behav Cogn Ther. (2021) 31:3–13. doi: 10.1016/j.jbct.2020.09.001

[3] Huke V Turk J Saeidi S Kent A Morgan JF. Autism spectrum disorders in eating disorder populations: a systematic review. Eur Eating Disord Rev. (2013) 21:345–51. doi: 10.1002/erv.2244 23900859

[4] Westwood H Mandy W Tchanturia K. Clinical evaluation of autistic symptoms in women with anorexia nervosa. Mol Autism. (2017) 8:12. doi: 10.1186/s13229-017-0128-x 28331571 PMC5356303

[5] Nazar BP Peynenburg V Rhind C Hibbs R Schmidt U Gowers S. An examination of the clinical outcomes of adolescents and young adults with broad autism spectrum traits and autism spectrum disorder and anorexia nervosa: A multi centre study. Int J eating Disord. (2018) 51:174–9. doi: 10.1002/eat.22823 29331075

[6] Leppanen J Sedgewick F Halls D Tchanturia K. Autism and anorexia nervosa: Longitudinal prediction of eating disorder outcomes. Front Psychiatry. (2022) 13:985867. doi: 10.3389/fpsyt.2022.985867 36213911 PMC9533087

[7] Tchanturia K Dandil Y Li Z Smith K Leslie M Byford S. A novel approach for autism spectrum condition patients with eating disorders: Analysis of treatment cost-savings. Eur Eating Disord Rev. (2021) 29:514–8. doi: 10.1002/erv.2760 32648631

[8] Tchanturia K Larsson E Adamson J. How anorexia nervosa patients with high and low autistic traits respond to group Cognitive Remediation Therapy. BMC Psychiatry. (2016) 16:1–7. doi: 10.1186/s12888-016-1044-x 27682072 PMC5041290

[9] Stewart CS McEwen FS Konstantellou A Eisler I Simic M. Impact of ASD traits on treatment outcomes of eating disorders in girls. Eur Eating Disord Rev. (2017) 25:123–8. doi: 10.1002/erv.2497 28058799

[10] Kinnaird E Norton C Stewart C Tchanturia K. Same behaviours, different reasons: what do patients with co-occurring anorexia and autism want from treatment? Int Rev Psychiatry. (2019) 31:308–17. doi: 10.1080/09540261.2018.1531831 30821179

[11] Brede J Babb C Jones C Elliott M Zanker C Tchanturia K. “For me, the anorexia is just a symptom, and the cause is the autism”: Investigating restrictive eating disorders in autistic women. J Autism Dev Disord. (2020) 50:4280–96. doi: 10.1007/s10803-020-04479-3 PMC7677288 32274604

[12] Babb C Brede J Jones CRG Elliott M Zanker C Tchanturia K. 'It's not that they don't want to access the support . . . it's the impact of the autism': The experience of eating disorder services from the perspective of autistic women, parents and healthcare professionals. Autism. (2021) 25:1409–21. doi: 10.1177/1362361321991257 PMC8264634 33588579

[13] Tchanturia K Smith K Glennon D Burhouse A. Towards an improved understanding of the anorexia nervosa and autism spectrum comorbidity: PEACE pathway implementation. Front Psychiatry. (2020) 11:640. doi: 10.3389/fpsyt.2020.00640 32733294 PMC7358367

[14] Li Z Halls D Byford S Tchanturia K. Autistic characteristics in eating disorders: Treatment adaptations and impact on clinical outcomes. Eur Eat Disord Rev. (2022) 30:671–90. doi: 10.1002/erv.2875 34850503

[15] Kinnaird E Norton C Tchanturia K. Clinicians' views on working with anorexia nervosa and autism spectrum disorder comorbidity: a qualitative study. BMC Psychiatry. (2017) 17:292. doi: 10.1186/s12888-017-1455-3 28797223 PMC5553805

[16] Adamson J Kinnaird E Glennon D Oakley M Tchanturia K. Carers’ views on autism and eating disorders comorbidity: qualitative study. BJPsych Open. (2020) 6:e51. doi: 10.1192/bjo.2020.36 32419683 PMC7331083

[17] O'Nions E Petersen I Buckman JE Charlton R Cooper C Corbett A. Autism in England: assessing underdiagnosis in a population-based cohort study of prospectively collected primary care data. Lancet Regional Health–Europe. (2023) 29:100626. doi: 10.1016/j.lanepe.2023.100626 37090088 PMC10114511

[18] Loomes R Hull L Mandy WPL. What is the male-to-female ratio in autism spectrum disorder? A systematic review and meta-analysis. J Am Acad Child Adolesc Psychiatry. (2017) 56:466–74. doi: 10.1016/j.jaac.2017.03.013 28545751

[19] Hull L Petrides KV Mandy W. The female autism phenotype and camouflaging: A narrative review. Rev J Autism Dev Disord. (2020) 7:306–17. doi: 10.1007/s40489-020-00197-9

[20] Lai MC Lombardo MV Ruigrok AN Chakrabarti B Auyeung B Szatmari P. Quantifying and exploring camouflaging in men and women with autism. Autism. (2017) 21:690–702. doi: 10.1177/1362361316671012 27899710 PMC5536256

[21] Allison C Auyeung B Baron-Cohen S. Toward brief “Red Flags” for autism screening: The Short Autism Spectrum Quotient and the Short Quantitative Checklist for Autism in toddlers in 1,000 cases and 3,000 controls [corrected]. J Am Acad Child Adolesc Psychiatry. (2012) 51:202–212.e207. doi: 10.1016/j.jaac.2011.11.003 22265366

[22] Westwood H Eisler I Mandy W Leppanen J Treasure J Tchanturia K. Using the autism-spectrum quotient to measure autistic traits in anorexia nervosa: A systematic review and meta-analysis. J Autism Dev Disord. (2016) 46:964–77. doi: 10.1007/s10803-015-2641-0 PMC4746216 26542816

[23] Kerr-Gaffney J Harrison A Tchanturia K. The social responsiveness scale is an efficient screening tool for autism spectrum disorder traits in adults with anorexia nervosa. Eur Eat Disord Rev. (2020) 28:433–44. doi: 10.1002/erv.2736 PMC8653883 32243021

[24] Kenny L Hattersley C Molins B Buckley C Povey C Pellicano E. Which terms should be used to describe autism? Perspectives from the UK autism community. Autism. (2016) 20(4):442–62. 10.1177/1362361315588200 26134030

[25] Smith KA Tchanturia K. Are huddles the missing PEACE of the puzzle in implementing clinical innovation for the eating disorder and autism comorbidity? Front Psychiatry. (2020) 11:593720. doi: 10.3389/fpsyt.2020.593720 33250797 PMC7674675

[26] Duggleby W Peacock S Ploeg J Swindle J Kaewwilai L Lee H. Qualitative research and its importance in adapting interventions. Qual Health Res. (2020) 30:1605–13. doi: 10.1177/1049732320920229 32458731

[27] Campbell M Moore G Evans RE Khodyakov D Craig P. ADAPT study: adaptation of evidence-informed complex population health interventions for implementation and/or re-evaluation in new contexts: protocol for a Delphi consensus exercise to develop guidance. BMJ Open. (2020) 10:e038965. doi: 10.1136/bmjopen-2020-038965 PMC7375505 32690750

[28] Braun V Clarke V. Using thematic analysis in psychology. Qual Res Psychol. (2006) 3:77–101. doi: 10.1191/1478088706qp063oa

[29] Braun V Clarke V. One size fits all? What counts as quality practice in (reflexive) thematic analysis? Qual Res Psychol. (2021) 18:328–52. doi: 10.1080/14780887.2020.1769238

[30] Tong A Sainsbury P Craig J. Consolidated criteria for reporting qualitative research (COREQ): a 32-item checklist for interviews and focus groups. Int J Qual Health Care. (2007) 19:349–57. doi: 10.1093/intqhc/mzm042 17872937

[31] PEACE Pathway. [Communication passport] (2020). Available online at: https://www.peacepathway.org/download/16.

[32] Tchanturia K. Supporting autistic people with eating disorders: A guide to adapting treatment and supporting recovery. London: Jessica Kingsley Publishers (2021).

[33] Li Z Holetic V Webb J Chubinidze D Byford S Tchanturia K. In-person and online sensory wellbeing workshop for eating disorders: updated case series. J Eating Disord. (2023) 11:117. doi: 10.1186/s40337-023-00834-8 PMC10347786 37443135

[34] Chubinidze D Li Z Slovak P Baudinet J Dufour E Tchanturia K. Introducing a smart toy in eating disorders treatment: a pilot study. Nutrients. (2024) 16(4), 467. doi: 10.3390/nu16040467 38398792 PMC10891985

[35] NICE. Autism spectrum disorder in adults: diagnosis and management. Natl Institute Health Care Excellence. (2012). 32186834

[36] Spain D Happé F. How to optimise cognitive behaviour therapy (CBT) for people with autism spectrum disorders (ASD): A Delphi study. J Rational-Emotive Cognitive-Behavior Ther. (2020) 38:184–208. doi: 10.1007/s10942-019-00335-1

[37] Spain D. Psychological therapies for adults with autism. New York, USA: Oxford University Press (2022). doi: 10.1093/med-psych/9780197548462.001.0001

[38] Walters S Loades M Russell A. A systematic review of effective modifications to cognitive behavioural therapy for young people with autism spectrum disorders. Review Journal of Autism and Developmental Disorders. (2016) 3:137–53.

[39] Lee SJ Altschul I Mowbray CT. Using planned adaptation to implement evidence-based programs with new populations. Am J Community Psychol. (2008) 41:290–303. doi: 10.1007/s10464-008-9160-5 18307029

[40] NHS England. Plan, Do, Study, Act (PDSA) cycles and the model for improvement. UK: NHS England (2021).

[41] von Thiele Schwarz U Aarons GA Hasson H. The Value Equation: Three complementary propositions for reconciling fidelity and adaptation in evidence-based practice implementation. BMC Health Serv Res. (2019) 19:1–10. doi: 10.1186/s12913-019-4668-y 31752846 PMC6873662

[42] Tchanturia K Lloyd S Lang K. Cognitive remediation therapy for anorexia nervosa: current evidence and future research directions. Int J eating Disord. (2013) 46:492–5. doi: 10.1002/eat.22106 23658098

[43] Schmidt U Wade TD Treasure J. The Maudsley Model of Anorexia Nervosa Treatment for Adults (MANTRA): development, key features, and preliminary evidence. J Cogn Psychother. (2014) 28(1). doi: 10.1891/0889-8391.28.1.48 32759130

[44] Lock J Le Grange D. Treatment manual for anorexia nervosa: A family-based approach. New York, USA: Guilford publications (2015).

[45] Duggleby W Williams A. Methodological and epistemological considerations in utilizing qualitative inquiry to develop interventions. Qual Health Res. (2016) 26:147–53. doi: 10.1177/1049732315590403 26063607

[46] Ashwood K Gillan N Horder J Hayward H Woodhouse E McEwen F. Predicting the diagnosis of autism in adults using the Autism-Spectrum Quotient (AQ) questionnaire. psychol Med. (2016) 46:2595–604. doi: 10.1017/S0033291716001082 PMC4988267 27353452

[47] Adamson J Brede J Babb C Serpell L Jones CRG Fox J. Towards identifying a method of screening for autism amongst women with restrictive eating disorders. Eur Eat Disord Rev. (2022) 30:592–603. doi: 10.1002/erv.2918 35791612 PMC9540024

[48] Russell G Stapley S Newlove-Delgado T Salmon A White R Warren F. Time trends in autism diagnosis over 20 years: a UK population-based cohort study. J Child Psychol Psychiatry. (2022) 63:674–82. doi: 10.1111/jcpp.13505 34414570

[49] Murphy CM Wilson CE Robertson DM Ecker C Daly EM Hammond N. Autism spectrum disorder in adults: diagnosis, management, and health services development. Neuropsychiatr Dis Treat. (2016) 12, 1669–86. doi: 10.2147/NDT PMC4940003 27462160

[50] Lipinski S Boegl K Blanke ES Suenkel U Dziobek I. A blind spot in mental healthcare? Psychotherapists lack education and expertise for the support of adults on the autism spectrum. Autism. (2022) 26:1509–21. doi: 10.1177/13623613211057973 PMC9344568 34825580

[51] NHS England. Autism waiting time statistics: autism statistics (2023). Available online at: https://digital.nhs.uk/data-and-information/publications/statistical/autism-statistics/april-2022-to-march-2023.

[52] National Autistic Society. Autism assessment waiting times (2023). Available online at: https://www.autism.org.uk/what-we-do/news/autism-assessment-waiting-times-2023#:~:text=Over%20140%2C000%20face%20waits%20for%20autism%20assessment&text=This%20is%20a%2040%25%20increase,referred%20and%20first%20being%20seen.

[53] Brede J Cage E Trott J Palmer L Smith A Serpell L. “We Have to Try to Find a Way, a Clinical Bridge”-autistic adults' experience of accessing and receiving support for mental health difficulties: A systematic review and thematic meta-synthesis. Clin Psychol Rev. (2022) 93:102131. doi: 10.1016/j.cpr.2022.102131 35180632

[54] Riese B Mukherjee RA. The experiences of autistic adults during the COVID-19 pandemic in the UK and implications for autism services development. Adv Autism. (2022) 8:343–53. doi: 10.1108/AIA-06-2021-0026

[55] Wigham S Ingham B Le Couteur A Wilson C Ensum I Parr JR. A survey of autistic adults, relatives and clinical teams in the United Kingdom: And Delphi process consensus statements on optimal autism diagnostic assessment for adults. Autism. (2022) 26:1959–72. doi: 10.1177/13623613211073020 PMC9597166 35168407

